# Upregulated desmin/integrin β1/MAPK axis promotes elastic cartilage regeneration with increased ECM mechanical strength

**DOI:** 10.7150/ijbs.83024

**Published:** 2023-05-21

**Authors:** Wei Zhang, Wei Lu, Qian Yu, Xia Liu, Haiyue Jiang

**Affiliations:** Plastic Surgery Hospital, Chinese Academy of Medical Sciences and Peking Union Medical College, Beijing 100144, PR China.

**Keywords:** auricular chondrocyte, tissue engineering, desmin, integrin β1, MAPK

## Abstract

Elastic cartilage tissue engineering is promising for providing available scaffolds for plastic reconstructive surgery. The insufficient mechanical strength of regenerative tissue and scarce resources of reparative cells are two obstacles for the preparation of tissue-engineered elastic cartilage scaffolds. Auricular chondrocytes are important reparative cells for elastic cartilage tissue engineering, but resources are scarce. Identifying auricular chondrocytes with enhanced capability of elastic cartilage formation is conducive to reducing the damage to donor sites by decreasing the demand on native tissue isolation. Based on the biochemical and biomechanical differences in native auricular cartilage, we found that auricular chondrocytes with upregulated desmin expressed more integrin β1, forming a stronger interaction with the substrate. Meanwhile, activated MAPK pathway was found in auricular chondrocytes highly expressing desmin. When desmin was knocked down, the chondrogenesis and mechanical sensitivity of chondrocytes were both impaired, and the MAPK pathway was downregulated. Finally, auricular chondrocytes highly expressing desmin regenerated more elastic cartilage with increased ECM mechanical strength. Therefore, desmin/integrin β1/MAPK signaling can not only serve as a selection standard but also a manipulation target of auricular chondrocytes to promote elastic cartilage regeneration.

## Introduction

Elastic cartilage tissue engineering is promising for providing scaffolds in multiple plastic surgeries by avoiding harm to donor sites. The mechanical properties of reconstructed tissue or organs are essential to maintain their shape and improve patients' experience. Suitable mechanical strength sustains the scaffold shape and avoids damage to native tissue 1, 2. The final mechanical properties are greatly impacted by regenerative tissue produced by reparative cells. Auricular chondrocytes are important reparative cells but their availability is greatly limited. As the main generator of the ECM, the biological activity of auricular chondrocytes is critical for the regeneration and maturation of elastic cartilage, which has not yet been investigated specifically. The biological properties of chondrocytes can be maintained within finite passages *in vitro* 3, 4. Moreover, the specific ability of chondrogenesis can be preserved by forming the corresponding cartilage matrix *in vitro* similar to their origin 5. Such characteristics can be found not only in different types of cartilage but also in different regions of certain irregular cartilage. For example, articular chondrocytes derived from different zones of articular cartilage are reported to behave differently with various chondrogenic abilities 6-9. In addition, chondrocyte bioactivity can be influenced by microstructural variations in the extracellular matrix (ECM) 10. Such heterogeneity has been applied in osteochondral tissue engineering to mimic gradient microstructure to induce osteochondral regeneration 11. The external ear has been distinguished by its intricate morphology and varied biomechanical properties across the auricular cartilage 12-15. The composition and arrangement of the ECM have been found to vary in different regions of auricular cartilage and evolve with age 16-19. Therefore, investigating the heterogeneity of the auricular cartilage microstructure and chondrocytes is of great significance in reparative cell selection and scaffold design to improve elastic cartilage regeneration.

The integrity and function of the construction unit, which consists of the cell, ECM, and their interaction, are vital factors that influence successful tissue regeneration 20. As the unit's center, cells can sense and respond to mechanical signals by altering their biological activities and synthesizing or degrading the ECM, thus impacting tissue formation and maturation 21, 22. Many failed tissue or organ developments have been linked to construction unit dysfunction 23, 24. The cytoskeleton-ECM coupled with interactions is a critical axis through which signals are transduced from the extracellular space to the intracellular space 25. Several cytoskeletons are reported to be associated with biomechanical transduction and corresponding cellular biochemical signaling 26. The expression and functions of interactions between cells and ECM, such as integrin and focal adhesion, are also impacted by environmental mechanical changes. Since different mechanical properties have been reported in native auricular cartilage 12, we investigated the basic construction unit relevant to such differences.

This study found that auricular cartilage close to the skull has increased mechanics, coupled with enhanced construction unit function. By comparing the expression profile, auricular chondrocytes with upregulated desmin-integrin β1-MAPK pathway regenerate more elastic cartilage with increased ECM mechanical strength, providing hints on cell selection and manipulation of auricular chondrocytes in elastic cartilage tissue engineering. Furthermore, decreased construction unit function was also found in microtia cartilage, indicating its importance for the maintenance of elastic cartilage mechanical strength.

## Methods and materials

### Tissue and cell isolation

Microtia and normal ear cartilage tissue were isolated from patients who underwent auricular reconstruction surgeries at the Plastic Surgery Hospital, Chinese Academy of Medical Sciences & Peking Union Medical College. Patient consent was obtained and the experiments were approved by the Institutional Research Ethics Committee Review Board. The animal experiments were approved by the Institutional Animal Care and Experiment Committee of Plastic Surgery Hospital, Chinese Academy of Medical Sciences & Peking Union Medical College. Ear cartilage was isolated from Bama miniature pigs (male, 6-12 months old) under general anesthesia. The skin and perichondrium were resected under aseptic conditions, leaving cartilage for cell isolation. The cartilage tissue was cut into pieces smaller than 1 mm × 1 mm and digested with 0.2% type II collagenase (Sigma) for 8 h at 37 °C in a shaker. The digested tissue was filtered using a 75-μm membrane and the collected cell suspension was centrifuged at 1000 rpm for 5 min. The cells were cultured and expanded using DMEM (10% FBS, 1% antibiotics) at 37 °C and 5% CO_2_. The experiments were conducted before passage 3.

### Aortic force microscopy (AFM)

AFM measurements were performed using a Dimension FastScan® AFM system (Bruker, Germany). Auricular cartilage with isolated skin and perichondrium was embedded in OCT compound, cryosectioned to a thickness of 10 μm and attached to a glass slide. The OCT was washed away with PBS before the AFM measurement. Samples were imaged in contact mode in air, and then indentation-type AFM measurements were conducted in PBS. A probe combining a silicon nitride cantilever with a cylindrical tip was used. The actual cantilever spring constant was measured using the thermo noise method after PBS addition. Under the guidance of an upright microscope, the intercellular ECM area was scanned by the probe for force curve collection and topography scanning. Topographical measurements were recorded at a resolution of 512 × 512 pixels^2^ with a line rate of 1 Hz. To assess the arrangement of fibers, the roughness and discrepancy were analyzed in a 1 μm^2^ area to decrease the influence of surface fluctuation. After topography scanning, PBS was added to the sample for elastic modulus measurement. To calculate the average stiffness, ten force-indentation curves were collected for each 1 × 1 μm^2^ area. Young's modulus was calculated with a modified Hertz model for a pyramidal indenter:

F = {(2 × tan α × E)/[π × (1-𝜈^2^)]}× δ^2^

where E is Young's modulus, 𝜈 is Poisson's ratio set as 0.5 for cartilage 27, and α is the tip half-opening angle. The data were analyzed using NanoScope Analysis 1.8 (Bruker, Germany).

### Traction force microscopy

Primary chondrocytes were isolated and manipulated for evaluation using traction force microscopy (TFM). First, cells were seeded on polyacrylamide gels containing red fluorescent beads. Two different concentrations of gels with stiffnesses of 10 kPa and 50 kPa were polymerized on confocal dishes, and 0.01% 0.5-μm red fluorescent carboxylate-modified beads were added to track displacement changes. A total of 1 × 10^4^ cells were seeded on the surface for measurement. To confirm the boundaries, phase-contrast images of cells were taken, whereas fluorescence images of red beads were captured prior to cell detachment to check for displacement changes. Finally, the beads were aligned using MATLAB to calculate the cellular interaction force with the substrate.

### Transmission electron microscopy

Auricular cartilage was cut into 1 mm × 1 mm pieces and fixed with 2.5% glutaraldehyde in 0.1 M sodium cacodylate buffer and stored at 4°C for transmission electron microscopy. The samples were freeze-dried, embedded in resin and sectioned into 100 nm ultrathin slices. The slices were coated by an ion sputtering instrument and observed using transmission electron microscopy (Microptik BV, Netherlands).

### Total RNA extraction and qPCR

Total RNA was extracted using TRIzol reagent (Thermo Fisher) following the manufacturer's instructions. Superscript II reverse transcriptase (Invitrogen) was used to generate cDNA from one microgram of mRNA. Real-time qPCR was performed using SYBR Green II mix on a LightCycle 480 Mix (Roche). Genes with quantification cycles (35 ≤ Ct ≤ 40) were regarded as having no expression and were discarded. The relative RNA expression was adjusted by GAPDH expression. Three duplicates with the same experimental conditions were obtained. The primer sequences are listed in Supplemental Table 2.

### RNA sequencing

Three adult Bama miniature pigs (6-12 months) were used as input material for the RNA sample preparations. Whole ear cartilage of three pigs was isolated and classified into lateral, middle, and medial areas. One piece of cartilage in each area was collected for total RNA extraction. Each group included three samples from three pigs. Briefly, mRNA was purified from total RNA using poly-T oligo-attached magnetic beads. After cDNA synthesis, cDNA fragment selection, and PCR amplification according to the manufacturer's protocols, the library was constructed. The obtained libraries were sequenced in 2 x 150 bp paired-end mode on an Illumina NovaSeq 6000 (Illumina, Inc., San Diego, CA, USA). After obtaining clean data from the raw data through in-house Perl scripts, the reference genome index was built using HISAT2 (v2.0.5), and paired-end clean reads were aligned to the reference genome using HISAT2. Eventually, RNA abundance was estimated with FeatureCounts (v1.5.0-p3). The gene fragments per kilobase million (FPKM) in each sample were calculated to identify differentially expressed genes (DEGs) with a cutoff value of p < 0.05 and |log2-fold change| > 1 via the DESeq2 R package 28. The biological functions of the DEGs were explored with Gene Ontology (GO) and Kyoto Encyclopedia of Genes and Genomes (KEGG) enrichment analyses 29, 30. Networks were generated in the Cytoscape software environment with the EnrichmentMap plugin using an uncorrected p value threshold of 0.005, an FDR cutoff of 0.1, and an overlap coefficient threshold of 0.1 31, 32. A protein-protein interaction (PPI) network was conducted using the Search Tool for Recurring Instances of Neighboring Genes (STRING) with a minimum required confidence score ≥ 0.7 33. Gene set enrichment analysis (GSEA) was performed using the clusterProfiler R package with c2.all.v7.0.entrez.gmt set as background (https://www.gsea-msigdb.org/gsea/msigdb). Reanalysis was further performed using screened DEGs with significantly increased expression from lateral and middle to medial areas.

### Biochemical analysis and histochemical staining

Auricular cartilage and GelMA constructs were prepared for ECM content assays. For the quantification of DNA and glycosaminoglycan (GAG) assays, samples were digested by proteinase K (0.5 mg/ml in PBS) at 56 °C for 16 h. The solubilized DNA and GAG were quantified using a PicoGreen dsDNA assay kit (Invitrogen) and GAG Alcian blue kit (GENMED) according to the manufacturer's instructions, respectively. The quantifications of collagen and elastin were performed using a hydroxyproline assay kit (Nan Jing Jiancheng Bioengineering Institute) and Fastin Elastin Assay kit (Biocolor), respectively.

For histochemical staining, auricular cartilage and GelMA constructs were fixed with 10% neutral buffered formalin overnight, dehydrated, and embedded in paraffin. Sections of 5 μm thickness were prepared for histochemical analysis. Following deparaffination, the sections were stained with H&E, Alcian Blue, and Verhoeff to visualize the general morphology and ECM components.

### 3D GelMA culture construct and migration test

For the 3D GelMA culture construct, a solution of 100 mg/mL GelMA (10% w/v) dissolved in DMEM containing 10% v/v LAP was prepared at 37 °C. Chondrocytes were mixed with GelMA solution at a density of 5 × 10^6^/mL at 37 °C. The photocrosslinkable hydrogel formulation was induced by exposure to blue light (wavelength: 405 nm; LED (Uvata Precision Optoelectronics Co., Ltd.); intensity: 20 mW/cm^2^) for 1 min at a distance of 10 cm. For the migration test in the GelMA construct, we selected the frequently used GelMA (10% w/v) and GelMA (5% w/v) as stiff and soft substrates, respectively. To simulate the varied tendency detected in native auricular cartilage, i.e., lateral < middle < medial, the intermediate GelMA (7.5% w/v) was set as the migration origin. GelMA constructs of 50 mg/mL (5% w/v) and 100 mg/mL (10% w/v) were prepared first as described above. Then, two semicircle constructs were placed in a round mold with a vacant center space. A mixed solution containing GelMA (7.5% w/v) and chondrocytes (1× 10^5^/mL) was added to the vacant center space. Then the formulation of the photocrosslinking recombinant constructs with gradient concentrations was induced by exposure to blue light (wavelength: 405 nm; LED (Uvata Precision Optoelectronics Co., Ltd.); intensity: 20 mW/cm^2^) for 1 min at a distance of 10 cm. The constructs (0.5 × 2 mm^3^) containing primary chondrocytes were cultured in DMEM supplemented with the addition of 10% FBS and 1% antibiotics. After 7 days of culture, 10% GelMA and 5% GelMA were isolated to analyze cell migration and chondrogenic marker expression.

### Western blot

Chondrocytes were washed twice using ice-cold PBS and lysed with RIPA buffer (Thermo Fisher) supplemented with proteinase inhibitor (Beyotime) for 15 min on ice. Protein concentration was assayed using a BCA protein assay kit (Beyotime) following the manufacturer's instructions. A total of 30 micrograms of protein was used for SDS‒PAGE electrophoresis and transferred onto a polyvinylidene fluoride membrane. The membrane was blocked with 5% milk in PBS with 0.05% Tween 20 (PBST) for 1 h at room temperature and then incubated with primary antibody overnight at 4°C. After washing three times with PBST, the membrane was incubated with HRP-conjugated secondary antibody for 1 h at room temperature. Bands were detected using a chemiluminescence ImageQuantTM LAS 4000 (GE), and the intensity of target bands was calculated using ImageJ.

### Flow cytometry

Passage 1 primary chondrocytes were digested using 0.25% trypsin and washed three times with PBS. The cell suspension was adjusted to 1 × 10^6^/mL and blocked using 5% BSA with 0.1% Triton X-100 for 1 h at room temperature. Next, the cells were incubated with the primary antibody for 1 h at 37 °C. After washing three times with PBS, the cells were incubated with fluorescence-labeled secondary antibody in darkness for 1 h at room temperature. Finally, the cells were washed twice with PBS and analyzed using a flow cytometer (BD Bioscience).

### Cartilage pellet culture

Auricular chondrocytes at passage 2 isolated from different ear regions were used for three-dimensional pellet culture. A cell suspension containing 5 × 10^5^ chondrocytes in a 15 mL polypropylene conical tube was centrifuged at 500 × g for 5 min. The supernatant was removed, and 2 mL DMEM containing 10% FBS was gently added to the tube without disturbing the cell sediment. Cells were maintained at 37 °C and 5% CO_2_. The cell pellet formed 72 h later and the medium was changed every 48 h thereafter.

### Immunofluorescence

Cells seeded on glass slides or cultured in GelMA constructs were fixed with 4% paraformaldehyde for 15 min and permeabilized with 0.1% Triton X-100 in PBS for 15 min. The samples were blocked with 5% BSA in PBS for 1 h at room temperature and then incubated with primary antibody overnight at 4°C. After washing three times with PBS, the samples were incubated with fluorescence-labeled secondary antibodies in darkness for 1 h at room temperature. Nuclei were stained using 4', 6'-diamidino-2-phenylindole (Beyotime) for 10 min. The stained cells on the glass slides were sealed using Prolong diamond antifade Mountant (Invitrogen). GelMA constructs were placed in the confocal dishes for observation. Samples were observed using confocal laser microscopy (Leica DM IRB). Images were processed and analyzed using ImageJ.

### Immunohistochemistry

Deparaffinized sections were treated with EDTA pH 9.0 in boiled water for 2 min and blocked with goat serum for 1 h at room temperature. After washing with PBS 3 times, the samples were incubated with primary desmin antibody (Proteintech, 16520-1-AP, 1:1000) overnight at 4°C. Then the sample was incubated with the corresponding secondary antibody at room temperature for 1 h. The DAB/AEC chromogen solution (Solarbio) covered the samples and was observed with a light microscope to identify the colored precipitate. The sample was washed with deionized H_2_O to terminate the reaction when obvious color precipitate appeared. Then the nuclei were counterstained with hematoxylin. Finally, the samples were dehydrated and sealed with neutral resin.

### Statistical analysis

The quantitative data are presented as the mean ± SEM for statistical analysis. Independent comparisons were made using an unpaired t test or Mann‒Whitney test. Significance was defined as ^*^p<0.05, ^**^p<0.01, ^***^p<0.001, ^****^p<0.0001. All statistical analyses were performed with GraphPad Prism version 8.

## Results

### Auricular cartilage was characterized by varied ECM mechanical strength and structural assemblies

**Figure 1:**
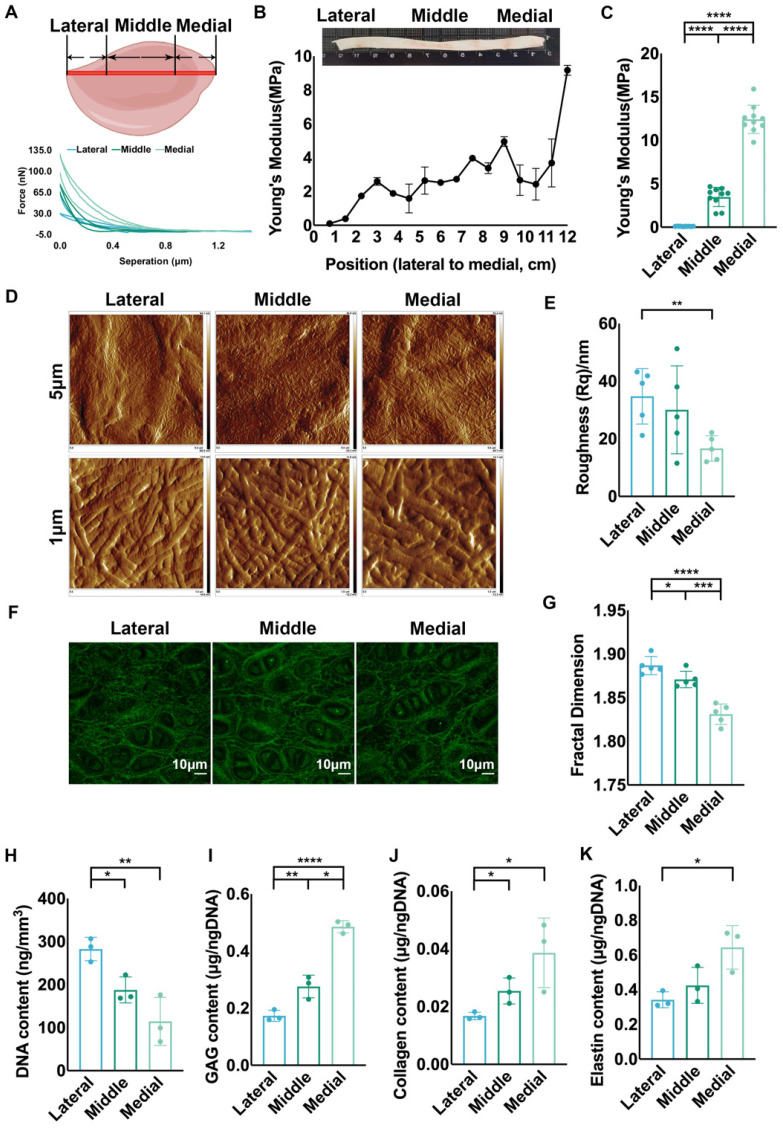
**Auricular cartilage was characterized by varied ECM mechanical strength and structural assemblies. A.** Native porcine auricular cartilage was divided into three parts: along the longest axis vertical to the skull, the outer ¼ area was lateral, the middle ½ area was middle, and the inner ¼ area was medial. Representative force curves scanned by AFM in three areas. Representative force curves for 16 positions with an interval of 0.75 cm for the AFM measurements. **B.** Statistical analysis of Young's modulus measured in 16 positions with an interval of 0.75 cm. **C.** Statistical analysis of the collective Young's modulus of positions located in three areas. (lateral vs. middle, ^****^p<0.0001; middle vs. medial, ^****^p<0.0001; lateral vs. medial, ^****^p<0.0001). **D.** Topography scanning of ECM fibers in three areas of native auricular cartilage. The scan area was set as 5 × 5 μm^2^ and 1 × 1 μm^2^. **E.** Statistical analysis of roughness Rq calculated based on a scanning area of 1 × 1 μm^2^ (lateral vs. medial, **p<0.01). **F.** Representative fluorescence images of elastic fibers in three areas of native auricular cartilage. **G.** Statistical analysis of fractal dimension calculated based on the intercellular elastic fibers in fluorescence images (lateral vs. middle, ^*^p<0.05; middle vs. medial, ^***^p<0.001; lateral vs. medial, ^****^p<0.0001). **H.** DNA content per volume in three areas of native auricular cartilage (lateral vs. middle, ^*^p<0.05; lateral vs. medial, ^**^p<0.01). Average **I.** GAG (lateral vs. middle, ^**^p<0.01; middle vs. medial,^ *^p<0.05; lateral vs. medial, ^**^p<0.01) **J.** collagen (lateral vs. middle, ^*^p<0.05; middle vs. medial,^ *^p<0.05), and **K.** elastin (lateral vs. medial, ^*^p<0.05) content produced per cell in three areas of native auricular cartilage.

Various macromechanical strengths have been reported in different areas of native auricular cartilage 34, and we measured ECM mechanics to explore the micromechanics that impact cellular biology. Based on previous studies showing that the area close to the skull has higher mechanical strength 12, 34, we chose the longest axis vertical to the skull as a reference for ECM mechanical analysis. Force curves were collected at sites with 0.75 cm intervals along the axis for stiffness analysis (Fig. 1A). The results showed that Young's modulus decreased gradually from the proximal to distal areas vertical to the skull (Fig. 1B). We classified the auricular cartilage into lateral, middle, and medial areas, and a significantly increased tendency was confirmed from the lateral to the medial areas (Fig. 1C). The topography of ECM fibers related to mechanics was scanned using AFM 35. More arranged ECM fibers were found in the middle and medial areas than in the lateral area (Fig. 1D, Fig. S1A). Statistical analysis showed that the roughness of the medial area was remarkably lower than that of the lateral area, reflecting more arranged ECM fibers (Fig. 1E, Fig. S1B). Meanwhile, the lateral area had more disoriented elastic fibers than the middle and medial areas (Fig. 1F), coupled with significantly higher fractal dimension (Fig. 1G). Given that the fractal dimension was influenced not only by the arrangement but also by the amount of ECM fibers, we measured the main components of the ECM in different areas. The results showed that the contents of GAG, collagen, and elastin were not significantly different across the areas in native auricular cartilage (Fig. S1C-E). Thus, the disarranged fibers might contribute to a higher fractal dimension in the lateral area. Moreover, we found that the cell density in the lateral area was significantly higher than that in the middle and medial areas (Fig. 1H, Fig. S1F). Since chondrocytes are the engine generating ECM, we investigated the average ECM production by chondrocytes across the different areas. The amounts of GAG, collagen, and elastin produced per cell were all the lowest in the lateral area (Fig. 1I-K). Collectively, auricular cartilage showed decreased ECM mechanics and irregular fiber arrangement in native auricular cartilage from the medial and middle to lateral areas, coupled with decreased average ECM production.

### Auricular cartilage with superior ECM mechanical properties has enhanced construction unit function

**Figure 2:**
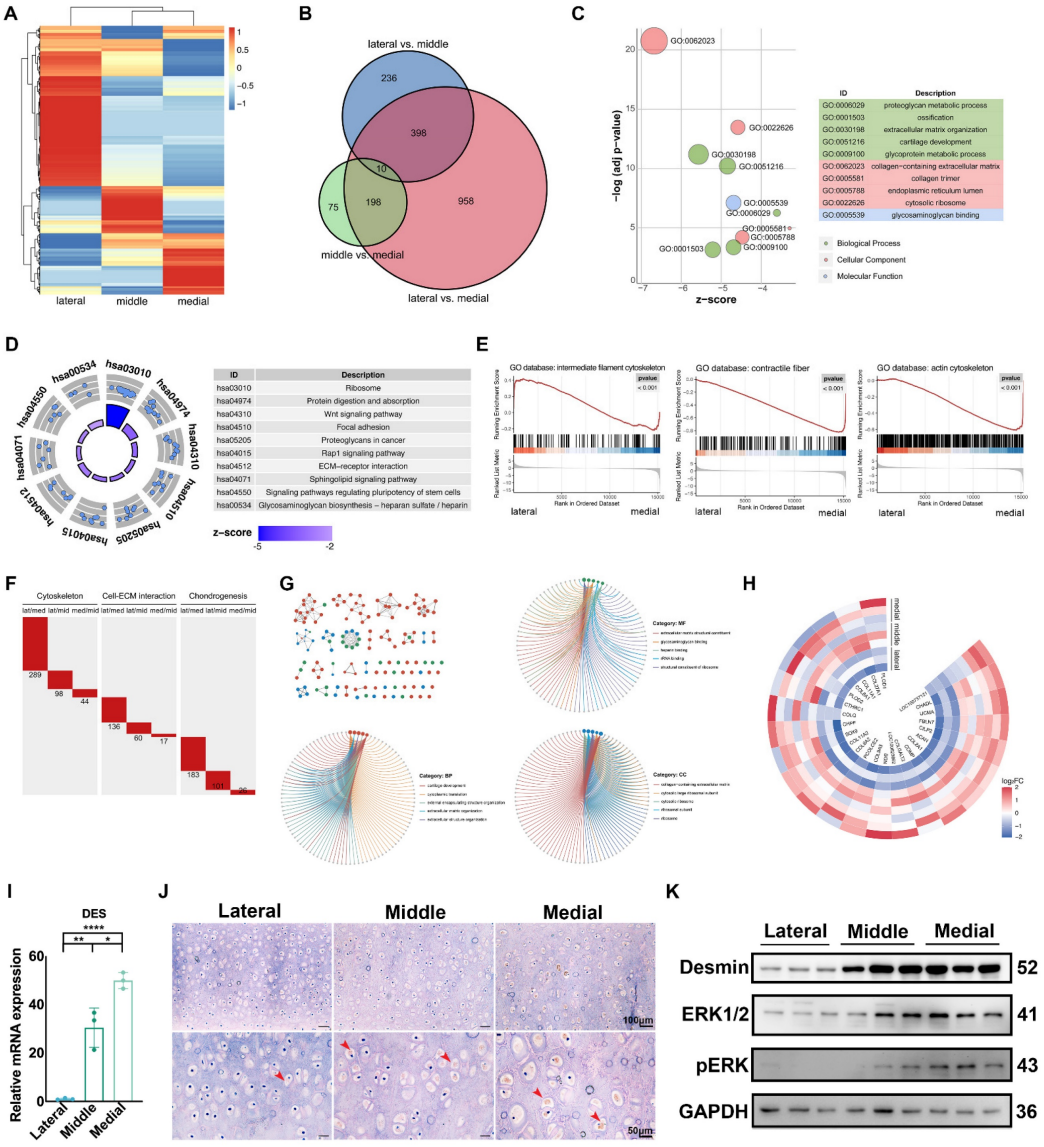
** Auricular cartilage with superior ECM mechanical properties has enhanced construction unit function. A.** Heatmap of gene expression in three areas of native auricular cartilage. **B.** Venn diagram of the differentially expressed genes (DEGs) among comparable groups of lateral vs. middle, middle vs. medial, and lateral vs. medial. **C.** GO analysis of DEGs between the lateral and medial areas. **D.** KEGG analysis of DEGs between the lateral and medial areas. **E.** GSEA analysis of DEGs between the lateral and medial areas. **F.** The number of DEGs in terms of cytoskeleton, cell-ECM interaction, and chondrogenesis among comparable groups of lateral vs. middle, middle vs. medial, and lateral vs. medial. **G.** DEGs related to cartilage ECM components with increased expression tendency from the lateral, middle, to medial areas. **H.** GO analysis of DEGs with increased expression tendency from the lateral to middle and medial areas. **I.** Relative mRNA levels of DES measured by qPCR (lateral vs middle, ^**^p<0.01; middle vs. medial, ^*^p<0.05; lateral vs. medial, ^****^p<0.0001). **J.** Immunohistochemistry analysis of desmin in the lateral, middle, and medial areas. (Red arrows indicate desmin) **K.** Western blot of desmin, ERK1/2, and pERK expression in chondrocytes isolated from three areas of native auricular cartilage.

We performed RNA sequencing (RNA-seq) on native auricular cartilage to investigate the underlying molecular mechanism related to ECM mechanics. The heatmap showed that the expression patterns in different areas were obviously separated (Fig. 2A). The number of DEGs between the lateral and medial areas was the largest (Fig. 2B, Fig. S2A), and these DEGs were selected for further analyses. GO analysis showed that DEGs were mainly enriched in ECM component structure and organization and cellular metabolic activities (Fig. 2C). In addition, KEGG analysis revealed that the activities of the ECM receptor, ECM synthesis, and Wnt pathways were significantly different between the lateral and medial areas (Fig. 2D). Moreover, GSEA indicated that genes related to the cytoskeleton, ECM production and organization were remarkably upregulated in the medial area (Fig. 2E, Fig. S2B). Therefore, different intensities of construction unit function were discovered in native auricular cartilage with varied ECM mechanics. Furthermore, we summed the number of DEGs related to the cytoskeleton, cell-ECM interactions, and chondrogenesis to assess the variance in construction unit function across areas. Consistent with the varied tendency in the whole transcriptome, the number of DEGs related to the construction unit was the highest between the lateral and medial areas, which had the largest mechanical difference (Fig. 2F).

### Auricular chondrocytes highly expressing desmin showed superior ECM production

We screened DEGs with an increased expression tendency from the medial and middle to lateral areas for analysis. GO analysis indicated that these DEGs were enriched in ECM synthesis and cell-ECM interactions (Fig. 2G). including multiple DEGs related to chondrogenesis (Fig. 2H). In addition, one critical intermediate cytoskeleton desmin was found to be significantly upregulated in the middle and medial areas (Fig. 2I, J). The shortage of desmin has been proven to be a critical pathogen in cardiac and skeletal diseases 36-38. All these diseases are characterized by cellular dysfunction in mechanical regulation or responses. In addition, the lack of desmin could also lead to dysfunction of nuclear activity via the connection to the lamina 39. We reanalyzed the RNA sequencing results and found that MAPK signaling was significantly different across auricular cartilage, which was also reported in normal and microtia cartilage 40. Additionally, the MAPK signaling pathway is known to be mechanically sensitive via integrins 41-43. Considering the mechanical difference existing in the auricular cartilage, we measured the activity of MAPK signaling in auricular chondrocytes driving from different areas. The phosphorylation of MAPK was increased along with upregulated desmin expression (Fig. 2K), suggesting a correlation between desmin and MAPK signaling activation.

### Auricular chondrocytes highly expressing desmin formed stronger interactions with the ECM by increasing integrin β1 expression

**Figure 3:**
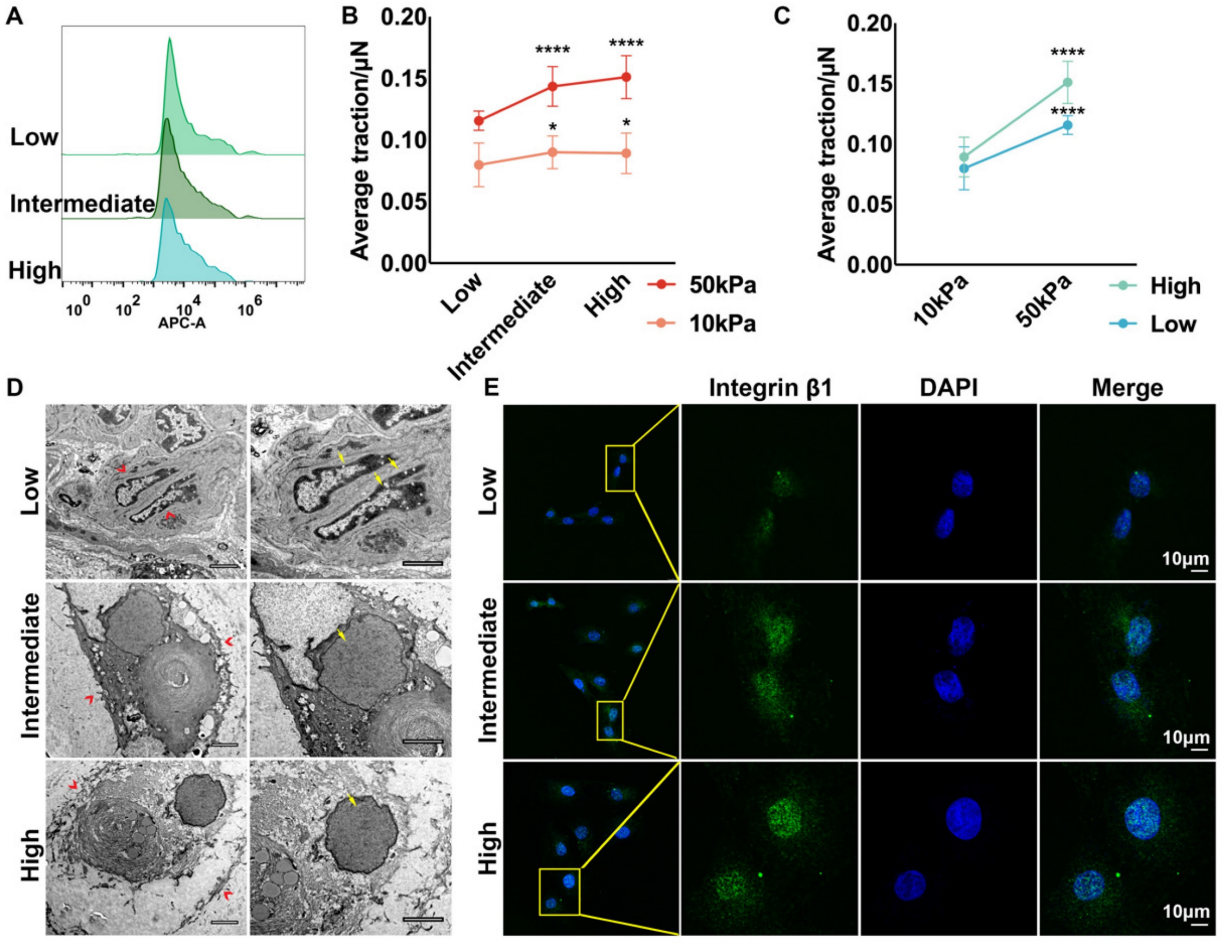
** Auricular chondrocytes highly expressing desmin formed stronger interactions with the ECM by increasing integrin β1 expression. A.** Flow cytometry of desmin expression in auricular chondrocytes isolated from three areas of native auricular tissue. **B.** Statistical analysis of the average traction force between chondrocytes with different desmin expression levels and substrates (50 kPa substrate: low vs intermediate, ^****^p<0.0001, low vs high, ^****^p<0.0001; 10 kPa substrate: low vs. intermediate, ^*^p<0.05, low vs. high, ^*^p<0.05). **C.** Statistical analysis of average traction force between chondrocytes and substrates with different stiffnesses (low: 10 kPa vs. 50 kPa, ^****^p<0.0001; high: 10 kPa vs. 50 kPa, ^****^p<0.0001). **D.** Representative TEM images of cells with different desmin expression levels in auricular cartilage (red arrows, cell-ECM connections; yellow arrows, nuclear chromatin). **E.** Representative immunofluorescence images of integrin β1 in chondrocytes with different desmin expression levels.

RNA-seq indicated that apart from the enhanced ECM organization and cytoskeleton, the interaction between ECM and chondrocytes was also stronger in the medial area, which is one of the essential contributors to tissue mechanics 44, 45. Auricular chondrocytes isolated from different areas were identified as having high, intermediate, and low desmin expression by flow cytometry (Fig. 3A). The interaction between the substrate and auricular chondrocytes with different desmin expression levels was measured using TFM. Chondrocytes with high desmin expression formed a stronger interaction force with stiff substrate (50 kPa) as well as soft substrate (10 kPa, Fig. 3B). Moreover, when chondrocytes with the same desmin expression were seeded on the soft and stiff substrates, the interaction force with stiff substrate was significantly larger than that with soft substrate (Fig. 3C). Interestingly, chondrocytes with higher desmin expression showed a larger increase in interaction force, indicating that desmin might be a mechanically sensitive marker. According to the SEM images, the protrusions of chondrocytes with low desmin expression into the ECM were the least, which indicated fewer connections between the chondrocytes and ECM (Fig. 3D). The integrin family has been reported to be sensitive to mechanical changes and to be able to mediate mechanical signal transduction, especially integrin β1, which was also downregulated in microtia cartilage 46-49. Immunofluorescence analysis showed that integrin β1 expression was significantly decreased in chondrocytes with low desmin expression (Fig. 3E). Integrin α4 is known to cooperate with integrin β1 as a complex to regulate cell-ECM interactions 50, so we also analyzed integrin α4 expression, but no significant difference was found (Fig. S2C). Therefore, integrin β1 was downregulated coupled with decreased chondrocyte-ECM interaction.

**Figure 4:**
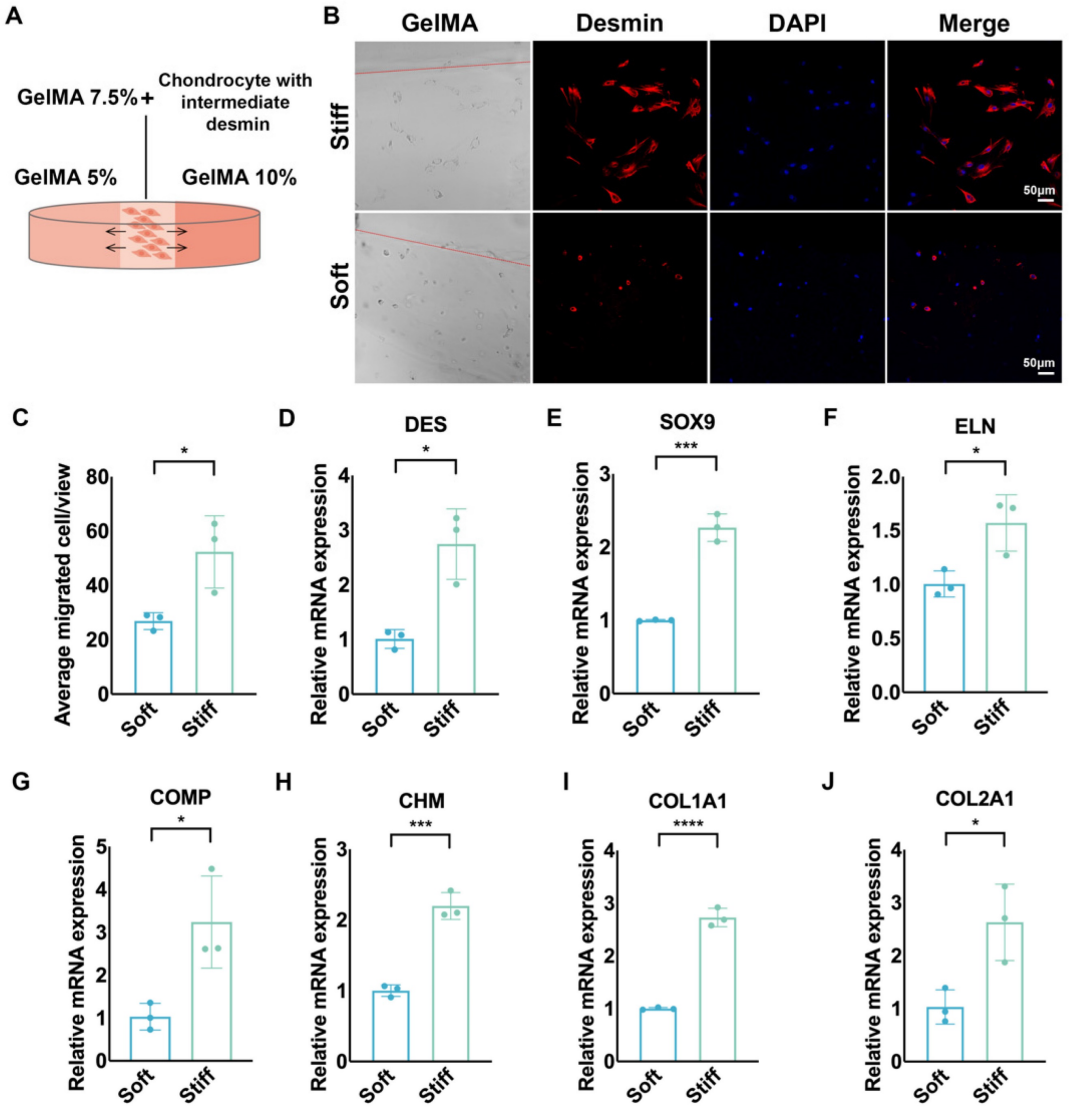
** Chondrocytes with high desmin expression demonstrated mechanical sensitivity and increased ECM production capability. A.** Schematic graph of chondrocyte migration to GelMAs with different stiffnesses. **B.** Representative immunofluorescence desmin staining of chondrocytes migrating into GelMAs with different stiffnesses. **C.** Statistical analysis of chondrocytes migrating into GelMAs with different stiffnesses (^*^p<0.05). Relative mRNA levels of **D.** DES (^*^p<0.05),** E.** SOX9 (^***^p<0.001),** F.** ELN (^*^p<0.05), **G.** COMP (^*^p<0.05), **H.** CHM (^***^p<0.001), **I.** COL1A1 (^****^p<0.0001), **J.** COL2A1 (^*^p<0.05) in chondrocytes migrating into GelMAs with different stiffnesses.

To further explore the mechanical sensitivity of desmin, we seeded chondrocytes with intermediate desmin expression in GelMA 7.5% (w/v), which was connected with GelMA 5% and GelMA 10% on the two sides by photocrosslinking (Fig. 4A). More cells migrated to stiff GelMA than to soft GelMA after seven days of culture (Fig. 4B, C). Meanwhile, chondrocytes migrating to stiff GelMA (10%) expressed more desmin than those migrating to soft GelMA (5%) (Fig. 4D), accompanied by upregulated gene expression of SOX9, ELN, COMP, CHM, COL1A1, and COL2A1 (Fig. 4E-J).

### Knockdown of desmin decreased the cell-ECM interactions, chondrogenesis, and mechanical sensitivity of auricular chondrocytes

**Figure 5:**
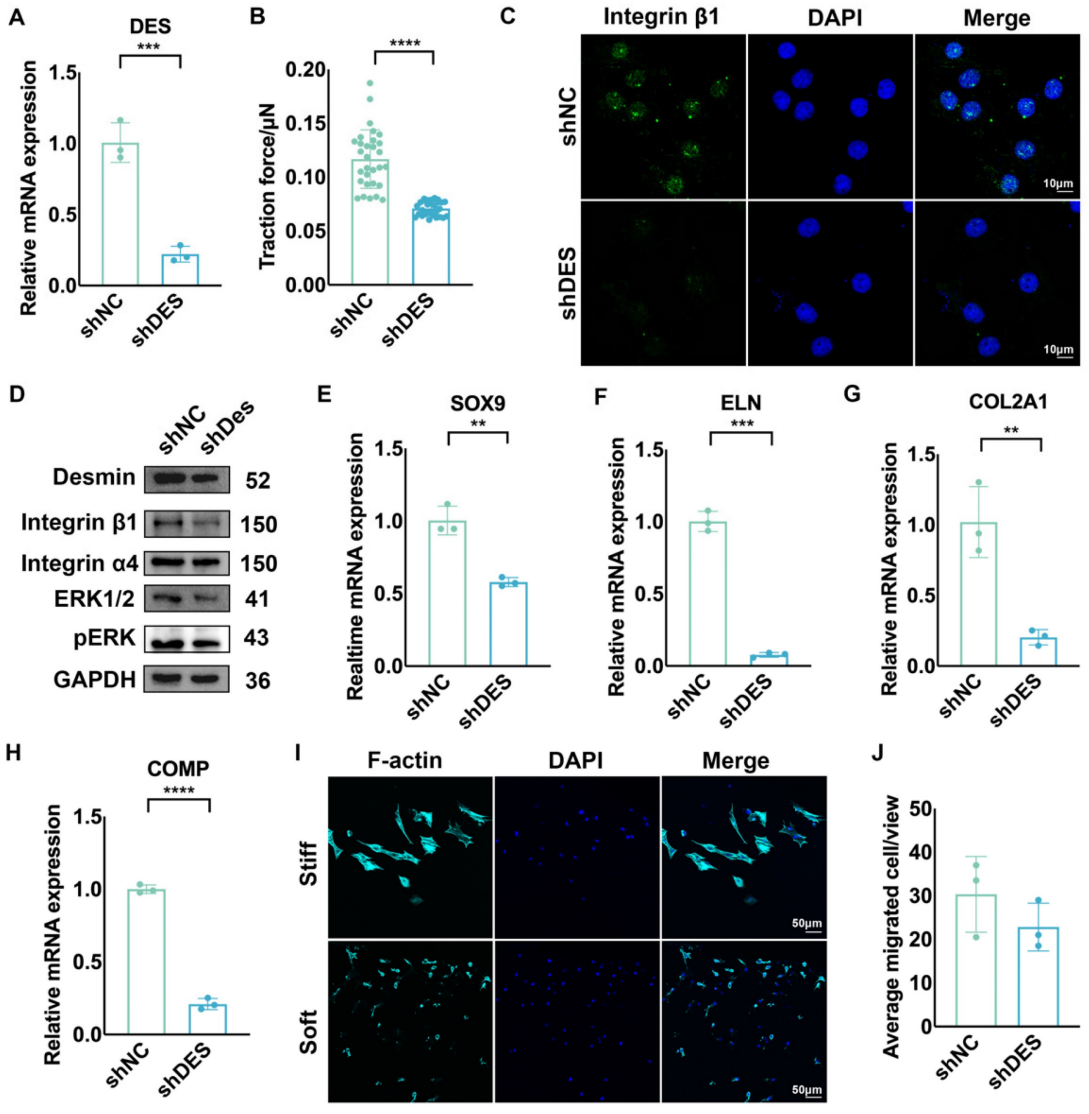
** Knockdown of Desmin decreased the mechanical sensitivity, cell-ECM interactions, and chondrogenesis of chondrocytes. A.** Identification of reduced DES mRNA levels after DES knockdown (^***^p<0.001). **B.** Average traction force between chondrocytes with DES knockdown and substrate (^****^p<0.0001). **C.** Representative immunofluorescence image of integrin β1 after DES knockdown. **D.** Western blot of integrin β1, integrin α4, ERK1/2, and pERK after DES knockdown. Relative mRNA levels of **E.** SOX9 (^**^p<0.01), **F.** ELN (^***^p<0.001), **G.** COL2A1 (^**^p<0.01), and **H.** COMP (^****^p<0.0001) after DES knockdown. I. Immunofluorescence of F-actin in chondrocytes with DES knockdown in the GelMA migration test. J. Statistical analysis of chondrocytes with DES knockdown migrating to GelMAs with different stiffnesses.

To confirm whether desmin interacts with ECM through integrin β1 and is related to the activation of the MAPK pathway, we knocked down desmin expression in chondrocytes, which was confirmed by mRNA and protein level measurements (Fig. 5A). Desmin knockdown significantly reduced the interaction force between chondrocytes and substrate (Fig. 5B). Additionally, integrin β1 expression was significantly reduced according to immunofluorescence (Fig. 5C). Western blot analysis also revealed that MAPK phosphorylation was suppressed after desmin knockdown as well as decreased integrin β1 expression (Fig. 5D). When the chondrogenesis ability was assessed, the mRNA levels of chondrogenesis-related genes, including SOX9, ELN, COL2A1, and COMP, significantly decreased following desmin knockdown (Fig. 5E-H). When seeded in GelMA with intermediate stiffness (7.5%), cells migrating to stiff and soft hydrogels showed no difference (Fig. 5I, J), indicating that mechanical sensitivity was impaired after desmin knockdown. Overall, auricular chondrocytes with high desmin expression showed a stronger interaction with ECM via integrin β1 and enhanced chondrogenesis capability with the activated MAPK signaling pathway.

### Decreased ECM mechanics and impaired construction unit function were also found in microtia cartilage

**Figure 6:**
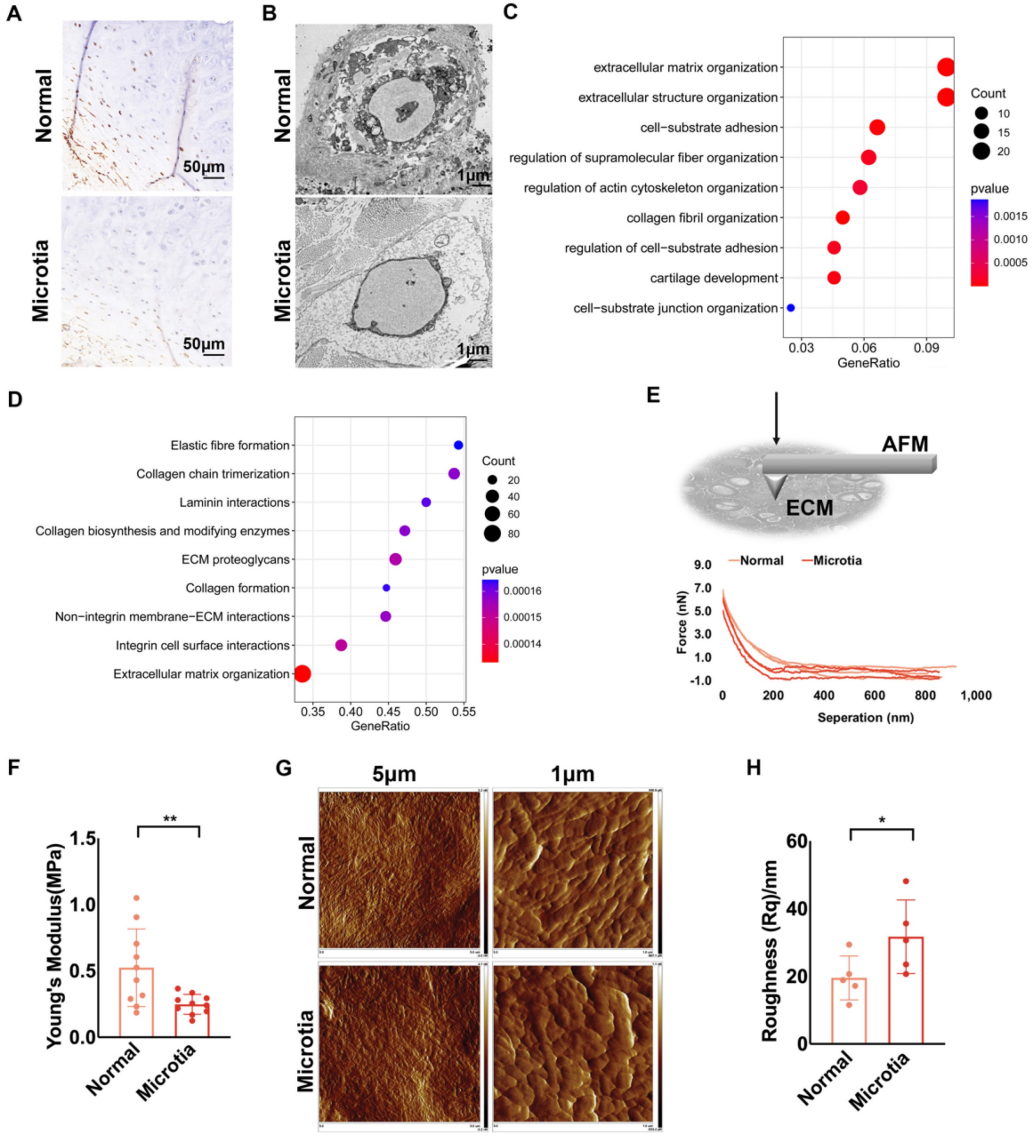
**Decreased ECM mechanics and impaired construction unit function were also found in microtia cartilage. A.** Representative immunohistochemical staining of desmin in normal and microtia cartilage isolated from the same patient (scale, 50 μm). **B.** Representative TEM images of normal and microtia auricular cartilage. **C.** GO analysis of differentially expressed proteins between normal and microtia cartilage. **D.** GSEA based on the Reactome database of differentially expressed proteins between normal and microtia cartilage (proteomics data from *Chen* et al*.*). **E.** Schematic graph: AFM measurement of intercellular ECM stiffness. Representative force curves scanned by AFM. **F.** Statistical analysis of the Young's modulus of normal and microtia auricular cartilage ECM (^**^p<0.01). **G.** Topography scanning of ECM fibers in normal and microtia auricular cartilage. The scan area was set as 5 × 5 μm^2^ and 1 × 1 μm^2^. **H.** Statistical analysis of roughness Rq calculated based on a scanning area of 1 × 1 μm^2^ (^*^p<0.05).

Microtia is a deformation of the external auricular cartilage that fails to form a normal external ear shape. We compared the characteristics of normal and microtia cartilage tissue from the same patient. According to immunohistochemical staining, chondrocytes in normal cartilage expressed more desmin than those in microtia cartilage (Fig. 6A, Fig. S3A). Fewer protrusions of chondrocytes into the ECM in the microtia cartilage suggested reduced cell-ECM interactions than in normal cartilage (Fig. 6B). Based on the transcriptomes of microtia and normal cartilage in the study by *Chen* et al*.*, MAPK signaling was downregulated in microtia cartilage. Furthermore, we reanalyzed the proteomics data of microtia and normal cartilage 40. Proteins related to ECM structure and cell-ECM interactions were differentially expressed between normal and microtia cartilage (Fig. 6C). Moreover, GSEA confirmed that cell-ECM interactions, especially those involving integrins, were downregulated in microtia cartilage (Fig. 6D). Force curves of ECM stiffness showed that the mean Young's modulus of microtia cartilage ECM was significantly lower than that of normal ear cartilage ECM (Fig. 6E, F). Considering the mechanical changes in cartilage with age 19, we compared the ECM stiffness of normal and microtia cartilage isolated from younger microtia patients. A similar ECM mechanical reduction was identified in microtia cartilage (Fig. S3B). The basic information about the patients is listed in Table S1. Topographic images showed less arranged and visible ECM fibers in microtia cartilage, coupled with higher roughness (Fig. 6G, H; Fig. S3C, D). Furthermore, more compacted pericellular and interwoven intercellular fibers were observed in normal cartilage than in microtia cartilage. Consistently, the fractal dimension index of intercellular elastic fibers was calculated to be larger in normal cartilage than in microtia cartilage, indicating more interwoven elastic fibers in normal cartilage (Fig. S3E). Collectively, the construction unit function and mechanical strength of microtia cartilage were impaired, indicating the correlation of construction unit function with elastic cartilage formation.

### Auricular chondrocytes highly expressing desmin regenerated more elastic cartilage with increased mechanical strength

**Figure 7:**
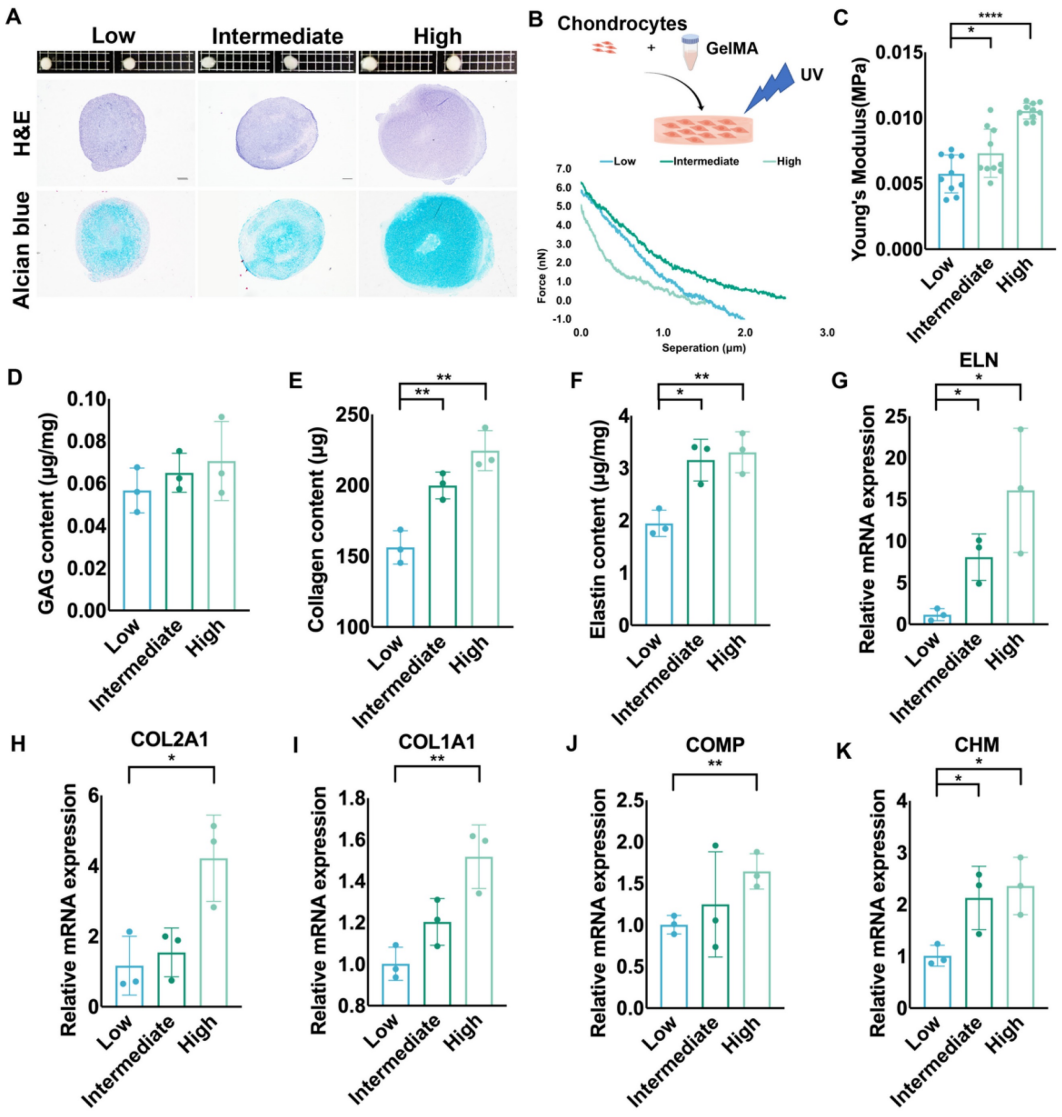
** Auricular chondrocytes highly expressing desmin regenerated more elastic cartilage with increased mechanical strength. A.** Histochemical staining of elastic cartilage pellets cultured by chondrocytes with different desmin expression levels. **B.** Schematic graph of 3D GelMA culture system establishment. Representative force curves of GelMA constructs seeded with chondrocytes with different desmin expression after one month of culture. **C.** Statistical analysis of Young's modulus of GelMA constructs (low vs. intermediate, ^*^p<0.05, low vs. high, ^****^p<0.0001). Assay of **D.** GAG, **E.** collagen (low vs. intermediate, ^**^p<0.01; low vs. high, ^**^p<0.01), and **F.** elastin (low vs. intermediate, ^*^p<0.05; low vs. high, ^**^p<0.01) production in GelMA constructs seeded with chondrocytes with different desmin expression after one month of culture. Relative mRNA levels of **G.** ELN (low vs. intermediate, ^*^p<0.05; low vs. high, ^*^p<0.05), **H.** COL2A1 (low vs. high, ^*^p<0.05) **I.** COL1A1 (low vs. high, ^**^p<0.01), **J.** COMP (low vs. high, ^**^p<0.01), and **K.** CHM (low vs. intermediate, ^*^p<0.05; low vs. high, ^*^p<0.05) in GelMA constructs seeded with chondrocytes with different desmin expression after one month of culture.

We cultured elastic cartilage pellets using auricular chondrocytes with different desmin expression levels to assess chondrogenesis. The size of pellets formed by auricular chondrocytes highly expressing desmin was the largest. Additionally, the amount of cartilage matrix in these pellets was also largest according to histochemical staining (Fig. 7A). We also cultured chondrocytes with different desmin expression levels in a GelMA three-dimensional culture to evaluate the regenerated ECM mechanics (Fig. 7B). After one month of culture, GelMA seeded with chondrocytes highly expressing desmin showed the highest Young's modulus (Fig. 7C), containing the most GAG, collagen and elastin production (Fig. 7D-F). Additionally, the cell number remaining in the culture system showed no significant difference among the three groups (Fig. S3F). Moreover, the RNA levels of chondrogenesis-related genes, such as ELN, COL1A1, COL2A1, COMP, and CHM were also highest in GelMA seeded with chondrocytes highly expressing desmin (Fig. 7G-K).

## Discussion

**Figure 8:**
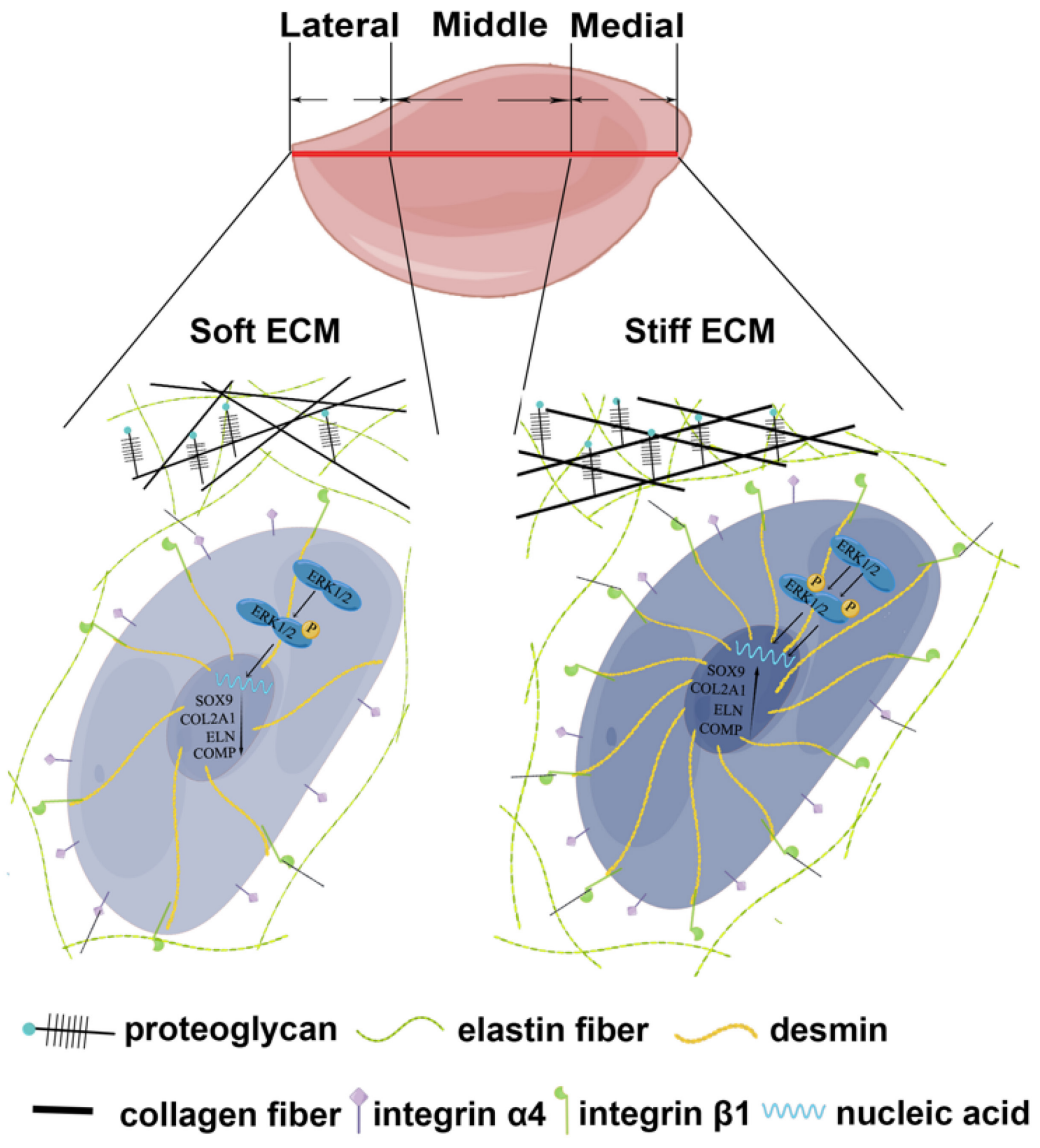
The schematic graph demonstrates that chondrocytes with high desmin expression showed a stronger interaction with ECM through integrin β1 and enhanced chondrogenesis capability with the activated MAPK signaling pathway.

Auricular chondrocytes are important reparative cells in elastic tissue engineering, but the resources are limited. The origin of reparative cells is the determinant of cellular behaviors 51, 52. Auricular cartilage showed heterogeneous biomechanical and biochemical properties. Auricular chondrocytes derived from medial and middle areas of native auricular cartilage showed upregulation of desmin/integrin β1/MAPK axis, coupled with superior chondrogenesis and enhanced mechanics of regenerated ECM (Fig. 8). Moreover, similar downregulation of signaling and impaired ECM mechanics were found in microtia cartilage with impairment, indicating the role of construction unit function in elastic cartilage formation. This evidence provides insight into the selection and manipulation targets of reparative cells to promote elastic cartilage regeneration.

The construction unit is the elementary block critical for the integrity and mechanical strength maintenance of tissue and organs 21, 53, 54. According to biomechanical and bioinformatic analyses, a reduction in construction unit function was observed in native auricular cartilage from the medial to the lateral areas. Moreover, we found that the downregulated desmin-integrin β1-MAPK axis was related to decreased mechanics across native auricular cartilage (ECM mechanics: medial > middle > lateral, ^****^p<0.0001). The activated MAPK signaling pathway and chondrogenesis can be induced by increased integrin β1 expression during cartilage formation 41, 42. In the present study, auricular chondrocytes with high desmin expression formed a stronger interaction with ECM by upregulating integrin β1, accompanied by activated MAPK pathway and enhanced chondrogenesis. Auricular chondrocytes also show a preference for stiff substrates with upregulated desmin expression, forming a positive feedback loop to promote elastic cartilage formation with enhanced mechanical eventually. Therefore, the desmin-integrin β1-MAPK axis can be regarded as a manipulated target for auricular cartilage regeneration. We confirmed that auricular chondrocytes in the areas close to skull express more desmin (medial > middle > lateral) and have superior chondrogenic capability. Therefore, isolation of reparative cells from specific parts can decrease injury to the donor site without impairing tissue regeneration.

The correlation between desmin and mechanics has also been reported in vessels. For example, the carotid artery lacking desmin showed less stiffness and connection with ECM, becoming more easily tortured under high blood pressure 55. Thus, chondrocytes with high desmin expression may be selected to promote elastic cartilage regeneration and increase mechanical strength. Moreover, genetic manipulation is increasingly used in tissue engineering to improve tissue regeneration. The safety of somatic genetic manipulation has been widely proven. In this regard, genetically overexpressed desmin may be one strategy for promoting cartilage regeneration in ear tissue engineering.

Interactions between the ECM and cells are critical to maintain the integrity and improve the mechanical strength of tissue and organs. Graceddo et al. reported that mechanical transduction could upregulate integrin 56. According to our study, chondrocytes with high desmin expression present a stronger interaction with ECM and more integrin β1 expression, resulting in higher mechanics and more cartilage cavity formation in 3D GelMA culture. Therefore, increased integrin β1 can facilitate the interaction force between the cell and ECM, which may eventually improve chondrocyte retention and mechanical strength. The surface modification of tissue-engineered scaffolds has been extensively studied. Since integrin β1 is a critical interactive molecule in auricular cartilage, surface modification using integrin β1 may also be an approach for scaffold optimization.

Scaffolds, serving as the main habitants of cells, should not only possess mechanical strength for shape maintenance but also provide a suitable environment for the proliferation and migration of cells. The microenvironment can influence cellular bioactivities and tissue regeneration. We found that auricular chondrocytes tend to migrate to spaces with higher stiffness, demonstrating a better performance of ECM production. Therefore, manipulating microenvironment mechanics could provide a suitable growing environment for cells, eventually realizing mechanical matching. One limitation of this study is that only the GelMA hydrogel system is utilized in this study, of which Young's modulus is lower than native cartilage tissue. However, as a biocompatible and easy-to-use component for scaffold complex preparation, the hydrogel is promising to be combined with other materials with high Young's modulus to establish scaffolds with controllable micromechanics, promoting chondrogenesis and integration into native tissue. Further experiments evaluating scaffold mechanical manipulation to promote cell migration and chondrogenesis *in vivo* can provide more insight into elastic cartilage tissue engineering.

## Conclusions

In summary, auricular chondrocytes with an upregulated desmin-integrin β1-MAPK axis are associated with superior chondrogenesis and enhanced ECM mechanical strength. This evidence provides insights into cell selection and manipulation targets in elastic cartilage tissue engineering.

## Supplementary Material

Supplementary figures and tables 1-3, tables 4-6 legends.

Supplementary table 4.

Supplementary table 5.

Supplementary table 6.

## Abbreviations

ECM: extracellular matrix
AFM: atomic force microscopy
TFM: traction force microscopy
GAG: glycosaminoglycan
DEG: differentially expressed gene
GSEA: gene set enrichment analysis
GO: gene oncology
KEGG: Kyoto Encyclopedia of Genes and Genomes
