# Supplementary material for: Upregulated desmin/integrin β1/MAPK axis promotes elastic cartilage regeneration with increased ECM mechanical strength
Source: Int J Biol Sci. 2023 May 21;19(9):2740–55. doi: 10.7150/ijbs.83024 (PMC10266073; doi:10.7150/ijbs.83024)
Supplement: Supplementary file 1 — Supplementary figures and tables 1-3, tables 4-6 legends. [file ijbsv19p2740s1.pdf]

Supplementary Figures

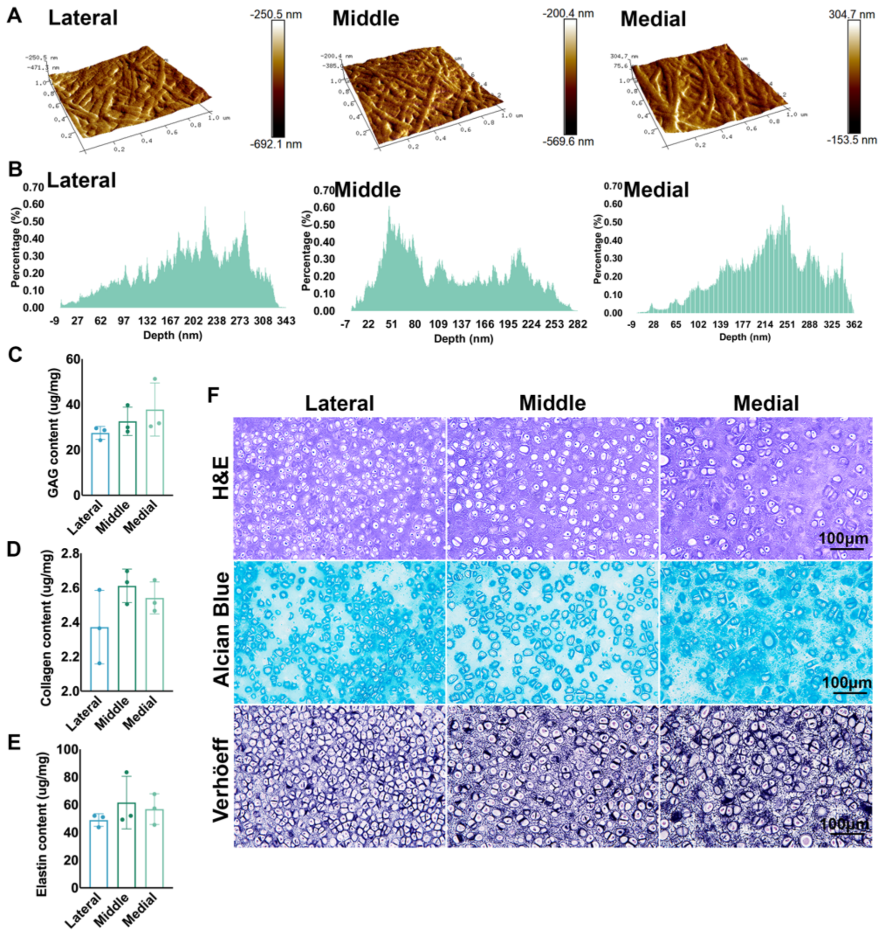

Supplementary Figure 1. A. Representative 3D topography images of ECM fibers across the different areas in native porcine auricular cartilage. **B.** Discrepancy of ECM fiber arrangement across the different areas in native porcine auricular cartilage. Assays of average **C.** GAG, **D.** collagen, and **E.** elastin contents per weight across the different areas in native porcine auricular cartilage. **F.** Histochemical staining of H&E staining, Alcian blue, and Verhöeff.

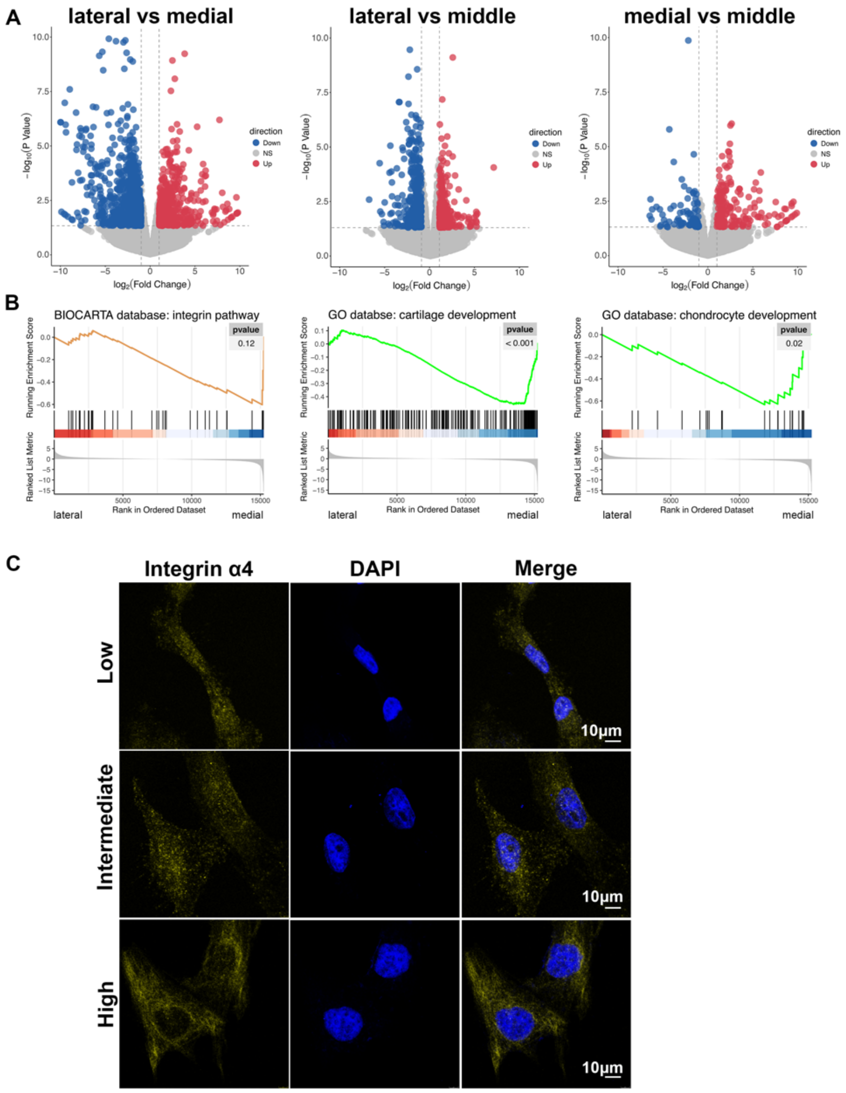

Supplementary Figure 2. A. Volcano plots of DEGs between the lateral vs middle, middle vs medial, and lateral vs medial comparisons. **B.** GSEA analysis of DEGs between the lateral and medial areas. **C.** Representative immunofluorescence images of integrin $\alpha 4$ in chondrocytes with different desmin expression levels.

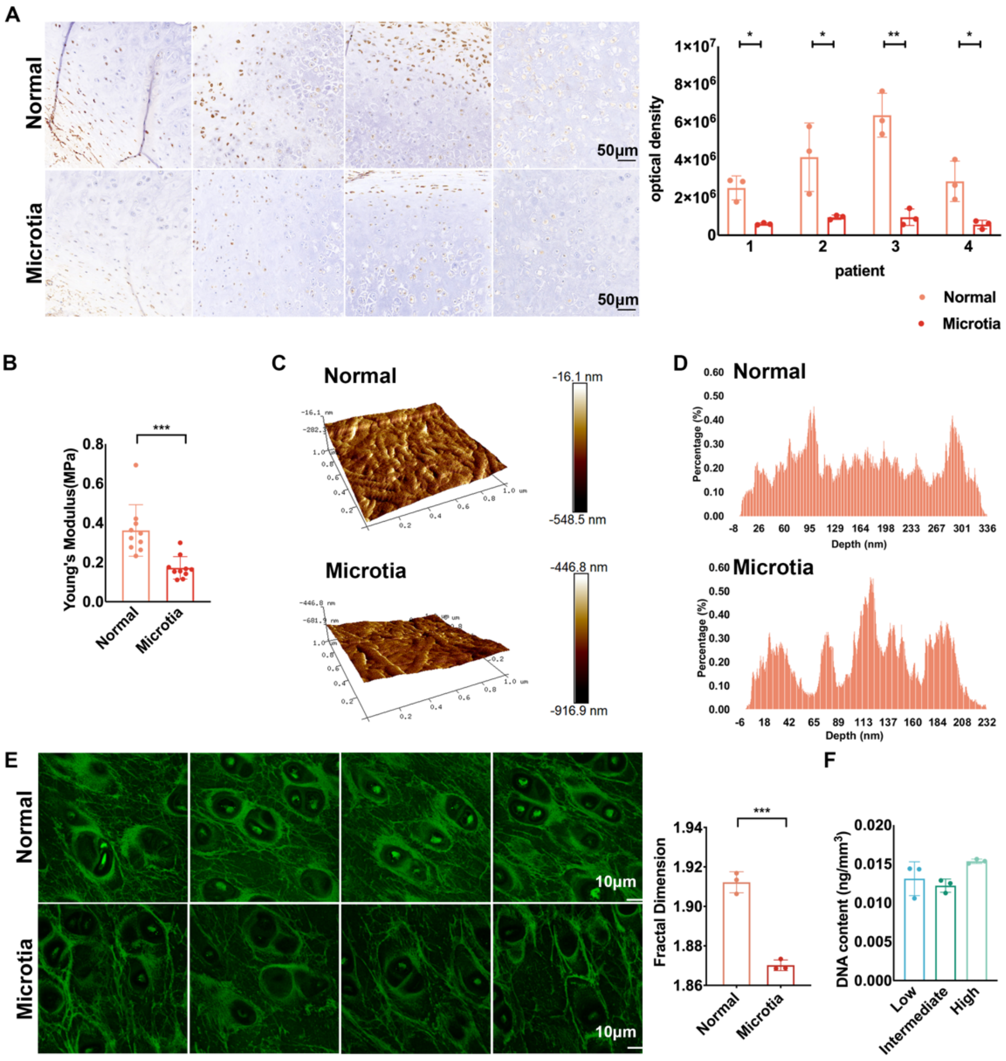

Supplementary Figure 3. A. Representative immunohistochemical images of desmin in normal and microtia auricular cartilage (n=4). Graph: statistical analysis of desmin expression in normal and microtia auricular cartilage in each patient. **B.** Statistical analysis of Young's modulus of normal and microtia auricular cartilage ECM in a younger patient (***$p<0.001$). **C.** Representative 3D topography images of ECM fibers in normal and microtia auricular cartilage. **D.** Discrepancy of ECM fibers in the normal and microtia auricular cartilage. **E.** Immunofluorescence images of normal and microtia auricular cartilage. Graph: statistical analysis of the fractal dimension calculated based on the intercellular elastic fibers in fluorescence images (***$p<0.001$). **F.** Assay of DNA content in 3D GelMA constructs.

Supplementary Table 1. Primer sequences (Forward and Reverse) for qRT–PCR.

Gene	Primer sequences
DES	F: CCTCAAGGGCACTAACGATTC
	R: GTTTCTCGGAAGTTGAGGGC
SOX9	F: GCAAACTCTGGAGACTGCTG
	R: TCTTCACCGACTTTCTCCGC
ELN	F: CCTATTTTCCCAGGTGGCGG
	R: ACTTTCTCTTCCGGCCACAG
COL1A1	F: CGATGGCTGCACGAGTCACAC
	R: CAGGTTGGGATGGAGGGAGTTTAC
COL2A1	F: TCCTGGTGAAGATGGTCGC
	R: AGCACCTGTCTCGCCATCT
COMP	F: GCCTGTGACGACGATGATG
	R: TTGTCTACCACCTTGTCCG
CHM	F: ACCAAGCAAAGCATCTCCTC
	R: GATTTCTTTCGGGTCGGTTG
ACAN	F: CCAGAATCTAGCAGGGAGTCATC
	R: AGGCAGAGGTGGCTTCAGTC
LOXL2	F: CCGACGACAACCCTTATTAC
	R: CGTGGATGCCTGGATGTAG
GAPDH	F: CTGCCCCTTCTGCTGATGC
	R: TCCACGATGCCGAAGTTGTC

Supplementary Table 2. Baseline information of microtia patients

Patient	Age (yrs)	Microtia	Sex
1	30	Left microtia	Male
2	8	Right microtia	Male
3	8	Right microtia	Male
4	9	Left microtia	Female

Supplementary Table 3. Antibodies

Antibody	Company	Cat No.	Work Ratio
Desmin Polyclonal antibody	Proteintech	16520-1-AP	WB 1:1000<br>IHC 1:1000<br>IF 1:200
ERK1/2 Polyclonal antibody	Proteintech	16443-1-AP	WB 1:1000
Phospho-ERK1/2 (Thr202/Tyr204) Polyclonal antibody	Proteintech	28733-1-AP	WB 1:1000
Mouse monoclonal [12G10] to Integrin beta 1	abcam	ab30394	WB 1:1000<br>IF 1:200
Rabbit monoclonal [EPR1355Y] to Integrin alpha 4/CD49D	abcam	ab81280	WB 1:1000<br>IF 1:200
Actin-Tracker Green	Beyotime	C1033	IF 1:200
GAPDH Monoclonal antibody	Proteintech	60004-1-Ig	WB 1:20000
Goat Anti-Mouse IgG H&L (HRP)	abcam	ab6789	WB 1:1000
Goat Anti-Rabbit IgG H&L (HRP)	abcam	ab6721	WB 1:1000<br>IHC 1:1000
Goat Anti-Mouse IgG H&L (Alexa Fluor® 488)	abcam	ab150113	IF 1:1000
Goat Anti-Rabbit IgG H&L (Alexa Fluor® 488)	abcam	ab150077	IF 1:1000
Goat Anti-Rabbit IgG H&L (Alexa Fluor® 647)	abcam	ab150079	IF 1:1000

	Flow Cyt 1:2000

Abbreviation: WB, western blotting; IF, immunofluorescence; Flow Cyt, flowcytometry; IHC, immunohistochemistry.

Supplementary Table 4. DEGs filtered by |log2|>1 of transcriptome data from *Chen* et al.

Supplementary Table 5. DEGs related to cytoskeleton, cell-ECM interactions, and ECM.

Supplementary Table 6. DEGs with increased expression tendency from the lateral to middle and medial areas in native auricular cartilage.
